# Risk Factors for Mortality in Patients With Strangulating Intestinal Obstruction Who Present With Septic Disseminated Intravascular Coagulation Prior to the Initiation of Treatment

**DOI:** 10.14740/gr2119

**Published:** 2026-02-28

**Authors:** En Amada, Yoshihiro Watanabe

**Affiliations:** a Department of Surgery, Sonoda-Daiichi Hospital, Adachi, Tokyo, Japan

**Keywords:** Strangulating intestinal obstruction, Disseminated intravascular coagulation, Prognostic risk factors

## Abstract

**Background:**

Strangulating ileus is a life-threatening surgical emergency characterized by intestinal ischemia and necrosis due to impaired blood flow and is frequently complicated by septic disseminated intravascular coagulation (DIC). Despite surgical and intensive care management, prognosis remains extremely poor. Early identification of prognostic factors associated with mortality is therefore crucial. This study aimed to identify early predictors of poor prognosis in patients with strangulating ileus complicated by septic DIC.

**Methods:**

We retrospectively analyzed 67 patients diagnosed with strangulating ileus at a single institution between 2020 and 2024, who subsequently developed septic DIC according to the Japanese Society for Emergency Medicine criteria and were treated with recombinant thrombomodulin (rTM). Patients with preoperative intestinal perforation, malignant tumors, or those receiving maintenance dialysis were excluded. Systemic inflammatory response syndrome (SIRS) score, Quick Sequential Organ Failure Assessment (qSOFA) score, Sequential Organ Failure Assessment (SOFA) score, DIC score, and white blood cell (WBC) count were evaluated preoperatively (Pre) and on the day after rTM administration (Day 1). Patients were divided into a survival group (S group) and a mortality group (M group), and variables were compared between groups. Univariate and multivariate logistic regression analyses were performed to identify independent prognostic factors.

**Results:**

Among the 67 patients, 38 survived and 29 died. On Day 1, SIRS scores, qSOFA scores, SOFA scores, and DIC scores were all significantly higher in the mortality group than in the survival group (P < 0.05). Mortality was also significantly higher in patients with a Day 1 WBC count < 8,000/µL and those with a Day 1 DIC score > 4. Multivariate logistic regression analysis identified a Day 1 WBC count < 8,000/µL (odds ratio (OR), 19.8; 95% confidence interval (CI), 3.64–72.6; P = 0.012) and a Day 1 DIC score > 4 (OR, 1.75; 95% CI, 1.27–9.34; P = 0.04) as independent predictors of mortality.

**Conclusions:**

In patients with strangulating ileus complicated by septic DIC, early leukopenia and persistently high DIC scores after rTM administration are independent poor prognostic factors. Early recognition of these indicators may allow prompt escalation of intensive care and contribute to improved outcomes in this highly lethal condition.

## Introduction

Strangulated intestinal obstruction is a critical surgical emergency that arises from compromised blood flow to the intestine, resulting in ischemia and subsequent necrosis. Immediate management is imperative to avert fatal complications. Common etiologies include postoperative adhesions, hernias, malignancies, and volvulus. Postoperative adhesions are prevalent in adults following abdominal surgery, while hernias often lead to development in underdeveloped regions [1–4]. The acute symptoms associated with this condition include severe abdominal pain, vomiting, and peritonitis. It is imperative that a prompt diagnosis be made in order to minimize morbidity and mortality. The diagnostic process entails a comprehensive evaluation, encompassing clinical assessment, laboratory tests, and computed tomography (CT) imaging. These diagnostic modalities yield indications such as diminished bowel wall enhancement and the presence of free abdominal fluid, which are suggestive of strangulation [5]. Urgent surgical intervention is imperative, with a focus on alleviating obstruction and resecting nonviable bowel segments. Preoperative stabilization through fluid resuscitation and electrolyte correction is essential [5]. The surgical interventions employed are contingent upon the findings and may encompass adhesiolysis, hernia repair, or bowel resection [3].

The prognosis is influenced by various factors, including delayed treatment, the presence of shock, and severe leukocytosis. Complications associated with sepsis can escalate, thereby increasing the risk of disseminated intravascular coagulation (DIC). DIC is characterized by systemic coagulation activation, leading to organ dysfunction and high mortality [6–9]. In the context of severe sepsis, DIC has been demonstrated to markedly elevate the incidence of multiple organ dysfunction syndrome and hospital mortality [10, 11]. Despite aggressive intervention strategies, outcomes for patients with sepsis complicated by DIC remain guarded, with mortality rates reported to approach 38% [11]. This underscores the necessity for enhanced diagnostic methodologies and therapeutic strategies to address the complex interplay between sepsis, DIC, and critical conditions such as strangulated intestinal obstruction.

Strangulating intestinal obstruction causes intestinal ischemia and increases intraluminal pressure, disrupting the intestinal barrier and allowing bacteria and endotoxins to translocate into the systemic circulation. This bacterial translocation can trigger sepsis, and septic DIC may develop early in the clinical course. Because septic DIC worsens organ dysfunction, early prognostic assessment during initial treatment is clinically important.

In order to enhance future treatment, it is imperative to identify these adverse prognostic factors and intervene against them promptly and comprehensively. However, such reports focusing on early-phase prognostic indicators in patients with strangulating intestinal obstruction complicated by septic DIC are scarce. The present study was therefore conducted to investigate poor prognostic factors identifiable during early treatment in this high-risk population.

## Materials and Methods

### Study design and patient selection

This retrospective observational study included patients diagnosed with strangulated intestinal obstruction at our institution between 2020 and 2024. Eligible patients were those diagnosed preoperatively with DIC using the Japanese Association for Acute Medicine (JAAM) DIC diagnostic criteria for sepsis [12] and who received recombinant thrombomodulin (rTM) during the treatment period.

The present study focused exclusively on patients treated with rTM because, according to the Japanese Society for Thrombosis and Hemostasis DIC treatment guidelines, rTM is the only drug recommended for administration to all patients diagnosed with DIC and represents the standard treatment for DIC in Japan [13].

### Exclusion criteria

Patients diagnosed preoperatively with intestinal perforation were excluded. Intestinal perforation was defined based on abdominal contrast-enhanced CT findings showing free air in the abdominal cavity or discontinuity of the intestinal wall. Additional exclusion criteria were as follows: 1) patients with chronic renal failure undergoing maintenance dialysis; 2) patients with a history of synchronous or metachronous malignancies (within 5 years), except for carcinoma *in situ*; and 3) patients with tumor lesions clearly suggestive of malignancy between the neck and pelvic floor identified on preoperative CT scans.

### Data collection and clinical variables

In addition to baseline patient characteristics, the following severity and clinical assessment scores were evaluated: the systemic inflammatory response syndrome (SIRS) score, Quick Sequential Organ Failure Assessment (qSOFA) score, Sequential Organ Failure Assessment (SOFA) score, and DIC score. Each score, along with the individual parameters required for their calculation, was measured at the time of preoperative DIC diagnosis (Pre) and 1 day after rTM administration (Day 1), which is equivalent to postoperative day 1.

Preoperative antithrombin activity was also collected for analysis.

### Outcome definition and group stratification

Patients were stratified into a survival group (S group) and a mortality group (M group) based on in-hospital outcomes. Survival outcome was defined as in-hospital survival. Patients who survived until hospital discharge were classified into the S group, whereas patients who died during the same hospitalization were classified into the M group, regardless of the length of overall survival.

### Statistical analysis

Continuous variables were assessed for normality and compared using an independent-samples *t*-test for normally distributed variables or the Mann–Whitney U test for non-normally distributed variables. Categorical variables were analyzed using the Chi-squared test.

Factors demonstrating significant differences between the S and M groups were further evaluated using logistic regression analysis. For continuous variables, receiver operating characteristic (ROC) curve analysis was performed prior to logistic regression, and the Youden index was calculated. The value corresponding to the maximum Youden index was defined as the cutoff value for each variable.

Logistic regression analysis was conducted to calculate odds ratios (ORs) with 95% confidence intervals (CIs), which were reported as OR (95% CI). Variables with a P value less than 0.05 in univariable analysis were included in the multivariable model. The goodness of fit of the logistic regression model was assessed using the Hosmer–Lemeshow test. Univariate analysis and stepwise multivariable analysis were performed to identify independent risk factors for mortality.

Because the SIRS score, qSOFA score, SOFA score, and DIC score represent independent severity assessment systems, each score was included separately in the analysis. Accordingly, the individual components of these scoring systems were also analyzed independently.

All statistical analyses were performed using SPSS version 28, and a P value less than 0.05 was considered statistically significant.

### Ethical considerations

This study was conducted in accordance with the Declaration of Helsinki and the ethical guidelines for research involving human subjects. The study protocol was approved by the Ethics Committee of Sonodakai and Sonoda Daiichi Hospital (approval number 172, approval date March 22, 2023).

This study was designed as an opt-out clinical research project; therefore, individual informed consent was not required. Patients were provided with the opportunity to refuse participation, and patient confidentiality and privacy were fully protected. All human organs and tissues used in this research were obtained in accordance with ethical and traceable protocols and adhered to standards established by the World Health Organization. The use of organs or tissues sourced from executed prisoners or prisoners of conscience was not involved. All procedures complied with relevant legislation and institutional regulations.

## Results

### Patient characteristics

A total of 67 patients with strangulating intestinal obstruction complicated by septic DIC were included in this study. Thirty-eight patients survived to hospital discharge (survival group), whereas 29 patients died during the same hospitalization (mortality group) (Table 1).

**Table 1:** Patient Background of the Study Subjects

	S group (n = 38)	M group (n = 29)	P value
Sex			
Male	35 (92.1%)	20 (69.0%)	0.11
Female	3 (7.9%)	9 (32.1%)	
Age (years)	68.5 (63–90)	78.5 (62–88)	0.32
ASA PS			
ASA I	0	0	NA
ASA II	0	0	
ASA III	0	0	
ASA IV	38 (100%)	29 (100%)	
ASA V	0	0	
ASA VI	0	0	
Body mass index (kg/m^2^)	20.2 (13.0–24.2)	19.9 (14.8–28.9)	0.89
Charlson Comorbidity Index	5 (0–8)	3 (0–7)	0.07
Hospital stay (days)	29.5 (15–92)	16 (4–161)	0.41
Overall survival (days)	358 (113–1,188)	34 (14–169)	< 0.001

Continuous variables were expressed as the median (minimum value–maximum value). ASA PS: American Society of Anesthesiologists Physical Status Classification System; S group: survival group; M group: mortality group; NA: not available.

Baseline demographic characteristics, including sex distribution, body mass index, and comorbidity burden assessed by the Charlson Comorbidity Index, did not differ significantly between the two groups. All patients were classified as American Society of Anesthesiologists physical status class IV. The mortality group was significantly older than the survival group. Length of hospital stay was shorter in the mortality group, reflecting early in-hospital death. Overall survival time differed markedly between the groups (Table 1).

### Surgical procedures and postoperative management

All patients underwent small bowel resection for strangulating intestinal obstruction (Table 2). The frequency of stoma creation, blood product transfusion, and adjunctive medical therapies did not differ significantly between the groups. However, patients in the mortality group more frequently required postoperative invasive respiratory support, including intermittent positive pressure ventilation, as well as renal replacement therapy. The use of endotoxin adsorption therapy showed a similar trend toward higher utilization in the mortality group (Table 2).

**Table 2:** Treatment Overview

	S group (n = 38)	M group (n = 29)	P value
Surgical procedure			
Partial small bowel resection	38 (100%)	29 (100%)	NA
Ileostomy	14 (36.8%)	8 (27.6%)	0.43
Jejunostomy or gastrostomy	18 (47.4%)	8 (27.6%)	0.09
rTM administration period	6 (1–10)	4 (1–16)	0.57
Blood transfusion			
Packed red blood cells	18 (47.4%)	16 (55.1%)	0.53
Fresh frozen plasma	19 (50.0%)	13 (44.8%)	0.68
Platelet concentrates	1 (2.6%)	2 (6.9%)	0.44
Glb	18 (47.4%)	7 (24.1%)	0.03
rAT-III	36 (94.7%)	28 (96.6%)	0.72
Steroid	8 (21.1%)	3 (10.3%)	0.23
Invasive treatment^a^	12 (31.6%)	7 (24.1%)	0.51
IPPV	11 (28.9%)	17(58.6%)	0.02
Intubation period	1 (1–152)	3 (1–44)	0.63
Tracheostomy	7/11 (58.3%)	7/17 (45%)	0.26
Renal replacement therapy	8 (21.1%)	20 (62.5%)	< 0.0001
Duration	3 (3–11)	12 (1–18)	0.36
PMX-DHP	8 (21.1%)	15 (46.9%)	0.01

^a^Additional invasive treatment performed after surgery. Continuous variables were expressed as the median (minimum value–maximum value). S group: survival group; M group: mortality group; rTM: recombinant thrombomodulin; rAT-III: recombinant antithrombin III protein; Glb: immune globulin infusion (human); IPPV: intermittent positive pressure ventilation; PMX-DHP: polymyxin B-immobilized fiber column direct hemoperfusion.

### Comparison of physiological and laboratory parameters

Physiological and laboratory parameters measured at the time of DIC diagnosis (Pre) and on the day after rTM administration (Day 1) are summarized in Table 3.

**Table 3:** Comparison of Physical and Laboratory Parameters Measured Before Treatment Initiation (Pre) and One Day After Treatment Initiation (Day 1)

	Pre			Day 1		
	S group (n = 38)	M group (n = 29)	P value	S group (n = 38)	M group (n = 29)	P value
GCS	14 (12–15)	10 (6–15)	0.12	15 (14–15)	12 (10–15)	0.003
Body temperature (°C)	38.3 (35.8–341.2)	37.2 (35.4–39.5)	0.36	36.7 (34.5–37.9)	36.7 (35.9–40.0)	0.67
Heart rate (beats/min)	127 (82–171)	119 (69–150)	0.41	91 (70–113)	119 (78–169)	0.27
Respiratory rate (/min)	28 (20–35)	28 (17–47)	0.9	20 (12–44)	23 (17–43)	0.27
Urine output (mL/day)	1,310 (690–4,700)	920 (690–1,410)	0.06	1,600 (10–3,800)	1,200 (280–2,245)	0.11
sNIBP (mm Hg)	102 (74–132)	89 (75–112)	0.08	118 (96–159)	100 (80–140)	0.36
MAP (mm Hg)	75 (62–90)	66 (60–84)	0.1	84 (71–100)	67 (55–105)	0.27
PaCO_2_ (Torr)	25.2 (22–45.9)	25.3 (12.8–48.0)	0.63	30.4 (22–60.9)	41.3 (32.0–64.7)	0.04
P/F ratio	350 (175–455)	212 (106–400)	0.2	328 (210–410)	235 (87–350)	0.03
WBC (/µL)	15,450 (8,600–44,900)	5,150 (1,600–27,200)	0.2	12,700 (5,200–47,600)	2,500 (1,600–19,900)	0.01
Platelet count (× 10^4^/µL)	12.4 (5.1–24.9)	11.7 (2.1–39.6)	0.83	9.4 (0.9–14.4)	10.2 (3.7–37.9)	0.48
Bil (mg/dL)	1.3 (0.5–4.3)	0.7 (0.3–3.1)	0.1	1.2 (0.5–3.6)	0.7 (0.3–6.9)	0.27
Cre (mg/dL)	1.09 (0.56–2.48)	1.48 (0.45–2.8)	0.52	0.99 (0.53–1.52)	1.11 (0.6–3.67)	0.6
PT-INR	1.24 (0.98–1.88)	1.39 (1.02–1.71)	0.46	1.26 (0.97–1.77)	1.37 (0.94–2.1)	0.89
FDP (µg/mL)	11.95 (5.9–17.5)	16.55 (7.7–57.7)	0.17	9.55 (5.9–22.4)	17.1 (6.2–44.6)	0.16
Antithrombin activity (%)	64.5 (39–80)	54.5 (41–70)	0.36	ND	ND	NA

Continuous variables were expressed as the median (minimum value–maximum value). S group: survival group; M group: mortality group; GCS: Glasgow Coma Scale; sNIBP: systolic noninvasive blood pressure; MAP: mean arterial pressure; PaCO_2_: arterial partial pressure of carbon dioxide; P/F ratio: Horowitz Index for Lung Function; WBC: white blood cell; Bil: serum total bilirubin level; Cre: serum creatinine level; PT-INR: prothrombin time-international normalized ratio; FDP: plasma fibrin and fibrinogen degradation products level; NA: not available; ND: not detected.

At the pretreatment assessment, most vital signs and laboratory values were comparable between the two groups, although the mortality group showed higher disease severity as reflected by the SOFA score.

On Day 1, patients in the mortality group demonstrated significantly worse neurological status, respiratory function, and circulatory parameters compared with the survival group. In particular, the mortality group exhibited lower Glasgow Coma Scale scores, higher arterial partial pressure of carbon dioxide, and lower P/F ratios (Horowitz Index for Lung Function). White blood cell (WBC) counts on Day 1 were markedly lower in the mortality group, whereas other routine laboratory parameters showed no significant differences (Table 3).

### Severity scores and prognostic factor analysis

Severity scores calculated before treatment initiation and on Day 1 are presented in Table 4. Although pretreatment SIRS, qSOFA, and DIC scores did not differ significantly between groups, the pretreatment SOFA score was significantly higher in the mortality group.

**Table 4:** Comparison of SIRS Score, qSOFA Score, SOFA Score and DIC Score Measured Before Treatment Initiation (Pre) and One Day After Treatment Initiation (Day 1)

	Pre			Day 1		
	S group (n = 38)	M group (n = 29)	P value	S group (n = 38)	M group (n = 29)	P value
SIRS score	3 (3–4)	4 (3–4)	0.15	1 (0–2)	2 (1–4)	0.03
qSOFA score	1 (1–3)	2 (2–3)	0.08	1 (0–2)	2 (1–3)	0.003
SOFA score	3 (1–6)	5 (1–9)	0.03	4 (1–7)	6 (2–16)	0.04
DIC score	4 (4–5)	4 (4–6)	0.2	2 (1–4)	5 (4–6)	< 0.001

Continuous variables were expressed as the median (minimum value–maximum value). S group: survival group; M group: mortality group; SIRS score: systemic inflammatory response syndrome score; qSOFA score: quick SOFA score; SOFA score: Sequential Organ Failure Assessment score; DIC score: disseminated intravascular coagulation diagnostic criteria for sepsis.

On Day 1, SIRS, qSOFA, SOFA, and DIC scores were all significantly higher in the mortality group, indicating persistent or worsening organ dysfunction despite treatment (Table 4).

Variables showing significant between-group differences were further analyzed using logistic regression models. ROC curve analysis was used to determine optimal cutoff values for each parameter. In multivariate analysis focusing on physiological and laboratory variables, a Day 1 WBC count < 8,000/µL was identified as an independent predictor of in-hospital mortality (OR, 19.8; 95% CI, 3.64–72.6; P = 0.012) (Table 5).

**Table 5:** Logistic Regression Analysis for Mortality Risk Factor Assessment Targeting Physical and Hematological Parameters

Inspection period	Univariate			Multivariate		
	Odds ratio	95% CI	P value	Odds ratio	95% CI	P value
Day 1						
GCS < 14	28.5	2.07–37.9	0.012			0.59
PaCO_2_ > 38.0 (Torr)	12.2	1.29–21.3	0.029			0.98
P/F ratio < 250	12.1	1.94–21.2	0.031			0.11
WBC < 8,000 (/µL)	63.3	3.32–119.4	0.006	19.8	3.64–72.6	0.012

GCS: Glasgow Coma Scale; PaCO_2_: arterial partial pressure of carbon dioxide; P/F ratio: Horowitz Index for Lung Function; WBC: white blood cell; CI: confidence interval.

In a separate multivariate model evaluating severity scores, a Day 1 DIC score > 4 was independently associated with increased mortality risk (OR, 1.75; 95% CI, 1.27–9.34; P = 0.04) (Table 6).

**Table 6:** Logistic Regression Analysis for Mortality Risk Factor Assessment Targeting SIRS Score, qSOFA Score, SOFA Score and DIC Score

Inspection period	Univariate			Multivariate		
	Odds ratio	95% CI	P value	Odds ratio	95% CI	P value
Pre						
SOFA score > 4			0.16			
Day 1						
SIRS score > 2			0.29			
qSOFA score > 2	1.88	1.03–15.22	0.04			0.15
SOFA score > 4			0.26			
DIC score > 4	2.31	1.78–31.82	0.02	1.75	1.27–9.34	0.04

Pre: before treatment initiation; Day 1: 1 day after treatment initiation; SIRS score: systemic inflammatory response syndrome score; qSOFA score: quick SOFA score; SOFA score: Sequential Organ Failure Assessment score; DIC score: disseminated intravascular coagulation diagnostic criteria for sepsis; CI: confidence interval.

## Discussion

This study identified two early indicators associated with in-hospital mortality in patients with strangulating intestinal obstruction complicated by septic DIC: a Day 1 WBC count < 8,000/µL and a Day 1 DIC score > 4. Day 1 severity scores (SIRS, qSOFA, SOFA, and DIC) were higher in non-survivors, indicating that persistent physiologic derangement after initiation of treatment reflected a worse clinical trajectory. These findings suggest that reassessment on Day 1 provides prognostically relevant information during the early phase of care.

A low Day 1 WBC count may indicate more severe immune dysregulation in septic DIC. In early sepsis, inflammatory mediators stimulate bone marrow release of neutrophils, resulting in leukocytosis; however, as sepsis progresses, apoptosis of immune cells increases and immune exhaustion develops, leading to leukopenia [14–16]. Large cohort studies have demonstrated that declining or persistently low WBC trajectories after initial treatment are associated with increased mortality in septic shock [14, 15]. Therefore, Day 1 leukopenia in the present cohort likely reflected advanced systemic inflammation and immune suppression during the early treatment period, which was associated with a higher risk of mortality.

A persistently high Day 1 DIC score may indicate ongoing coagulopathy despite treatment initiation. DIC scoring systems quantify the severity of coagulation abnormalities and organ dysfunction, and failure of early improvement reflects inadequate response to therapy [10, 11]. Previous studies evaluating JAAM-based DIC criteria have shown that higher scores and lack of score improvement after treatment initiation are associated with increased mortality in septic DIC [11, 12, 17, 18]. Accordingly, the association between a Day 1 DIC score > 4 and mortality in the present study supports the concept that failure of early DIC improvement reflects ongoing systemic coagulopathy and worsened prognosis.

Several physiologic variables demonstrated consistent directional differences between survivors and non-survivors on Day 1. Survivors tended to show more stable neurologic status and respiratory function, as reflected by higher Glasgow Coma Scale scores and better oxygenation indices. These trends suggest less severe organ dysfunction in survivors, even when some individual variables did not reach statistical significance. Because the sample size was limited, statistical power to detect modest differences was restricted, and absence of statistical significance does not necessarily indicate absence of clinically meaningful differences.

This study has several limitations. First, the retrospective single-center design limits generalizability, and external validation in larger cohorts is required. Second, treatment strategies for septic DIC in Japan, including rTM, differ from those used in other regions [13]. Therefore, the prognostic performance of the identified indicators should be evaluated in different treatment settings. Third, the JAAM-based DIC criteria used in this study differ from the International Society on Thrombosis and Hemostasis overt DIC criteria, so extrapolation to cohorts defined by other diagnostic frameworks should be performed cautiously [9, 10].

In conclusion, reassessment on Day 1 identified leukopenia (WBC < 8,000/µL) and persistent coagulopathy (DIC score > 4) as early indicators associated with mortality in patients with strangulating intestinal obstruction complicated by septic DIC. These indicators are readily available during early treatment and may facilitate prompt identification of high-risk patients and timely escalation of intensive care.

## Data Availability

The data supporting the findings of this study are available from the corresponding author upon reasonable request.
